# Climate change, allergy and asthma, and the role of tropical forests

**DOI:** 10.1186/s40413-017-0142-7

**Published:** 2017-03-07

**Authors:** Gennaro D’Amato, Carolina Vitale, Nelson Rosario, Herberto Josè Chong Neto, Deborah Carla Chong-Silva, Francisco Mendonça, Josè Perini, Loraine Landgraf, Dirceu Solé, Mario Sánchez-Borges, Ignacio Ansotegui, Maria D’Amato

**Affiliations:** 1 Division of Respiratory and Allergic Diseases, Department of Respiratory Diseases, High Specialty Hospital A. Cardarelli, Napoli, Italy; 2 School of Specialization in Respiratory Diseases, University of Napoli Federico II, Napoli, Italy; 3 Department of Medicine and Surgery, University of Salerno, Salerno, Italy; 4 Federal University of Paraná, Curitiba, Brazil; 5 Board President of ASBAI-Brazilian Association of Allergy, Curitiba, Brazil; 6 Allergist Presidente of Paranà ASBAI, Curitiba, Brazil; 7 Department of Pediatrics, Federal University of São Paulo-Escola Paulista de Medicina, São Paulo, Brazil; 8 Allergy and Clinical Immunology Department, Centro Médico Docente La Trinidad and Clínica El Avila, Caracas, Venezuela; 9 Department of Allergy & Immunology, Hospital Quirón Bizkaia, Carretera Leioa-Unbe 33 bis 48950, Erandio, Spain; 10 First Division of Pneumology, High Speciality Hospital “V. Monaldi” and University “Federico II” Medical School, Napoli, Italy

**Keywords:** Respiratory allergy, Bronchial asthma, Climate change, Air pollution and respiratory diseases, Greenhouse gas emissions, Anthropogenic emissions of CO_2_, Interaction between climate change and allergy, Deforestation and climate change

## Abstract

**Background:**

Tropical forests cover less than 10 per cent of all land area (1.8 × 107 km^2^) and over half of the tropical-forest area (1.1 × 107 Km^2^) is represented by humid tropical forests (also called tropical rainforests). The Amazon basin contains the largest rainforest on Earth, almost 5.8 million km^2^, and occupies about 40% of South America; more than 60% of the basin is located in Brazil and the rest in Bolivia, Colombia, Ecuador, French Guiana, Guyana, Peru, Suriname and Venezuela.

Over the past decade the positive role of tropical rainforests in capturing large amounts of atmospheric carbon dioxide (CO_2_) has been demonstrated. In response to the increase in atmospheric CO_2_ concentration, tropical forests act as a global carbon sink.

**Main body:**

Accumulation of carbon in the tropical terrestrial biosphere strongly contributes to slowing the rate of increase of CO_2_ into the atmosphere, thus resulting in the reduction of greenhouse gas effect. Tropical rainforests have been estimated to account for 32–36% of terrestrial Net Primary Productivity (NPP) that is the difference between total forest photosynthesis and plant respiration. Tropical rainforests have been acting as a strong carbon sink in this way for decades.

However, over the past years, increased concentrations of greenhouse gases, and especially CO_2_, in the atmosphere have significantly affected the net carbon balance of tropical rainforests, and have warmed the planet substantially driving climate changes through more severe and prolonged heat waves, variability in temperature, increased air pollution, forest fires, droughts, and floods. The role of tropical forests in mitigating climate change is therefore critical. Over the past 30 years almost 600,000 km^2^ have been deforested in Brazil alone due to the rapid development of Amazonia, this is the reason why currently the region is one of the ‘hotspots’ of global environmental change on the planet.

**Conclusion:**

Deforestation represents the second largest anthropogenic source of CO_2_ to the atmosphere, after fossil fuel combustion. There are many causes of deforestation, including socioeconomic and natural factors, such as clear-cutting for agriculture, ranching and development, unsustainable logging for timber, as well as droughts, fires and degradation due to climate change. About natural causes of forest degradation, in the context of the Amazon, the major agent of change in the forest ecosystem would most likely be decreased dry-season precipitation. Of the 23 global climate models employed by the Intergovernmental Panel on Climate Change (IPCC) in their 2007 report, 50–70% predict a substantial (above 20%) reduction of dry-season rainfall in eastern Amazonia under mid-range greenhouse gas emissions scenarios, 40% in central Amazonia and 20% in the west. While annual carbon emissions from fossil-fuel combustion have been continually increasing since 1960s, historical trends of deforestation and associated carbon emissions have remained poorly understood.

## Background

Climate change represents a massive threat to global health, affecting local and national food supplies, air and water quality, weather, economics and many other critical health determinants [1, 2]. Air pollution is closely associated with climate change [1–4]. Over the last 50 years global earth’s temperature has markedly risen [1]. Most of the observed increase in globally averaged temperatures is very likely due to the observed increase in anthropogenic greenhouse gas concentrations, as stated in the Working Group I Report of the Intergovernmental Panel on Climate Change [1]. The key determinants of greenhouse gas emissions are energy production, transportation, agriculture, food production and waste management, and attempts at mitigating climate change will need to address each of these. A huge increase in carbon dioxide (CO_2_) concentrations during the last decades has been experienced [1]. CO_2_ is the most important anthropogenic greenhouse gas, and its atmospheric concentration has increased from a pre-industrial value of about 280 ppm to 379 ppm in 2005 [1]. About 75% of the anthropogenic CO_2_ emissions to the atmosphere during the past 20 years resulted from fossil fuel burning; most of the rest resulted from changes in land use, especially deforestation [1]. The same trend occurred for the other prevalent anthropogenic greenhouse gases: methane (CH_4_), and nitrous oxide (N_2_O) [1]. However, it is important to consider that after CO_2_ emissions are reduced and atmospheric concentrations stabilize, surface air temperature continues to rise slowly for a century or more. Furthermore, rising temperatures contribute to the elevation of the concentrations of ozone (due to more sunlight and higher temperature) and particulate matter (due to wildfire, droughts, desertification, sandstorms and an increased use of coal-fired power to produce energy for cooling) at ground level [1, 2].

A growing body of evidence indicates that climate change has a strong impact on respiratory health, particularly on respiratory allergic diseases [1–6].

Many measures to reduce greenhouse gas emissions may have positive benefits for health. According to the intergovernmental panel on climate change (IPCC), it is necessary to reduce the anthropogenic emissions of CO_2_; in this regard deforestation represents the second largest anthropogenic source of carbon dioxide to the atmosphere, after fossil fuel combustion [7]. The role of forest, particularly of rainforest of Amazon basin (the largest rainforest on Earth), in mitigating climate change is therefore critical.

This article aims to provide evidence to stimulate the debate on the impact of climate change on respiratory health and on the contribution of forests of Brazil in mitigating climate change.

### The effect of climate changes on pollen allergy and respiratory allergic diseases

A body of evidence suggests that major changes involving the atmosphere and the climate, including global warming induced by human activity, have an impact on the biosphere and human environment [1, 2].

A summary of the potential health effects due to climate change is presented in Table 1.

**Table 1:** Potential health effects of climate change

Climate events	Agriculture, forestry	Human health impact
Heavy precipitation events: frequency increases over most areas	Damage to crops; soil erosion, inability to cultivate land, water logging of soils; Adverse effects on quality of surface and groundwater; contamination of water supply	Deaths, injuries, infectious diseases, allergies and dermatitis from floods and landslides
Area affected by drought	Land degradation, lower yields/crop damage and failure; livestock deaths; land degradation; More widespread water stress	Increased risk of food and water shortage; increased risk of water- and food-borne diseases; cardiovascular disorders
Number of intense tropical cyclones	Damage to crops; wind throw of trees; Power outages cause disruption of public water supply	Increased risk of water- and food-borne diseases; asthma
Incidence of extreme high sea level	Salinization of irrigation and well water; Decreased freshwater availability due to saltwater intrusion	Increase in stress-related disease; other allergic conditions

Studies on the effects of climate changes on respiratory allergy are still lacking and current knowledge is provided by epidemiological and experimental studies on the relationship between asthma and environmental factors, eg, meteorological variables, airborne allergens and air pollution. Climate change is correlated with allergens for several reasons [8, 9]:

1. increase and faster plant growth;
2. increase in the amount of pollen produced by each plant;
3. increase in the amount of allergenic proteins contained in pollen,
4. increase in the start time of plant growth and therefore the start of pollen production and
5. earlier and longer pollen seasons.

An earlier start and peak of the pollen season is more pronounced in species that start flowering early in the year. Moreover, plants flower earlier in urban areas than in the corresponding rural areas with earlier pollination of about 2–4 days. Pollen counts could rise due to multiple mechanisms such as increased ambient carbon dioxide levels [10], increased temperature or earlier spring seasons [11]. With warming over the longer term, changing patterns of plant habitat and species density are likely, with gradual movement northward in the Northern Hemisphere, and further south in the Southern Hemisphere [12]. The change in land use might also play a relevant role, especially for some important allergenic species, such as grasses. However, since most data come from the analysis of distribution of airborne pollen, these findings are potentially biased by the occurrence of long and medium distance transport episodes of allergenic pollen [13, 14].

Pollen allergy is frequently used to study the interrelationship between air pollution and allergic respiratory diseases (rhinitis and asthma).

Epidemiologic studies have demonstrated that urbanization, high levels of vehicle emissions and westernized lifestyle are correlated with an increase in the frequency of pollen-induced respiratory allergy in people who live in urban areas compared to those who live in rural areas [15]. Studies on plant responses to elevated CO_2_ concentrations indicate that plants exhibit enhanced photosynthesis and reproductive effects and produce more pollen. Wayne et al. observed that a doubling of the atmospheric CO_2_ concentration stimulated ragweed-pollen production by 61%. [11]. Furthermore, ragweed pollen collected along high-traffic roads showed a higher allergenicity than pollen sampled in vegetated areas, and it is probably due to traffic related pollution. Climate change may also affect the release and atmospheric dispersion of pollen [16]. The overall impact will be an altered pollen season timing and load, and hence change in exposure.

One of the effects of climate change is an increasing frequency and intensity of floods and cyclones. An example of how this effect can threaten respiratory health is “Thunderstorm related asthma” [17]. Actually, thunderstorms occurring during the pollen season have been observed to induce severe asthma attacks in pollen-allergic patients [15–17]. Associations between thunderstorms and asthma morbidity have been identified in multiple locations around the world [17–19]. The most prominent hypotheses for thunderstorm-related asthma are linked with bioaerosols, and involve the role of rainwater in promoting the release of respirable particulate matter [17, 20].

After hydratation and rupture by osmotic shock during the beginning of a thunderstorm, pollen grains release part of their cytoplasmic content into the atmosphere, including inhalable, allergen-carrying paucimicronic particles such as starch granules and other cytoplasmic components [17, 20].

In summary the occurrence of these epidemics is closely linked to thunderstorms; the thunderstorm-related epidemics are limited to late spring and summer when there are high levels of airborne pollen grains; there is a close temporal association between the arrival of a thunderstorm, a major rise in concentration of pollen grains and the onset of asthma epidemics. As a consequence, subjects affected by pollen allergy should be alert to the danger of being outdoors during a thunderstorm in the pollen season.

### Interaction between climate change and urban air pollution

Climate change, coupled with air pollutant exposures, may have potentially serious adverse consequences for human health.

Some air pollution-related episodes of asthma exacerbations are due to climatic factors that favour the accumulation of air pollutants at ground level, and some cities are continuously affected by pollution caused by motor vehicles [21, 22]. Furthermore, it is also important to consider that worldwide, hundreds of thousands of hectares of woods are destroyed each year by fire, thus millions of tons of CO_2_ are producted, playing a role in the greenhouse effect [23–25].

Studies have demonstrated some effects of ozone over respiratory symptoms, acute decreases in lung function, increased airway responsiveness, airway injury and inflammation and systemic oxidative stress [26–29]. Gent et al. [26] examined the simultaneous effects of ozone and fine particulate matter (PM _2.5_) at levels below EPA standards on daily respiratory symptoms and rescue medication use among children with asthma. Ozone level (but not PM_2.5_) was significantly associated with respiratory symptoms and rescue medication use among children using maintenance medication. A 50 parts per billion (ppb) increase in 1-h ozone was associated with increased likelihood of wheeze (by 35%) and chest tightness (by 47%) [26]. The highest levels of ozone (1-h or 8-h averages) were associated with increased shortness of breath and rescue medication use [26].

One of the mechanisms whereby air pollutants can induce asthma is the interaction with allergen-carrying paucimicronic particles derived from plants [30]. The paucimicronic particles, pollen-originated or not, are able to reach peripheral airways with inhaled air, inducing asthma in sensitized subjects. Air pollution—in particular particulate matter (PM), and diesel exhaust particulate (DEP), ozone, nitrogen dioxide and sulfur dioxide — have been shown to have an inflammatory effect on the airways of susceptible subjects, causing increased permeability, easier penetration of allergens into the mucus membranes, and easier interaction with cells of the immune system [30]. There is also evidence that predisposed subjects have increased airway reactivity induced by air pollution and increased bronchial responsiveness to inhaled allergens [31]. By attaching to the surface of pollen grains and plant-derived particles of paucimicronic size, air pollutants could modify not only the morphology of these antigen-carrying agents but also their allergenic potential. In addition, by inducing airway inflammation, which increases airway permeability, pollutants overcome the mucosal barrier and could be responsible for “priming” the allergen-induced responses of pollinosis in allergic and atopic individuals.

The relationship between exposure to air pollution and the development of allergic respiratory diseases has been investigated in several studies, however there is still much to understand.

Nicolai and von Mutius carried out a study on this topic in reunified Germany. The prevalence of asthma and allergic disorders was assessed in East Germany and in West Germany [32]. In East Germany the main sources of air pollution were the industries and private coal burning for heating purposes, differently in in West Germany where traffic-related air pollutants and NO_2_ exposure were prevalent [32]. The authors thus analyzed the impact of different environmental and social conditions on the development of allergies in two genetically homogeneous populations. The results showed that hay fever, skin test reactivity to common aeroallergens and asthma were considerably more prevalent in West Germany as compared to East Germany [32].

Recently a systematic review and a meta-analysis of birth cohort studies have shown that increased longitudinal childhood exposure to PM_2.5_ and black carbon was associated with increasing risk of subsequent asthma in childhood [33]. Also, early childhood exposure to traffic-related air pollution was associated with development of asthma across childhood up to 12 years of age [33]. Increasing exposure to PM_2.5_ was associated with sensitization to both aero- and food allergens [33].

### How to reduce air pollution and global warming: the role of Brazilian forests and their message to the planet

Tropical forests cover less than 10% of all land area (1.8 × 107 Km^2^) [34] and over half of the tropical-forest area (1.1 × 107 Km^2^) is represented by humid tropical forests (also called tropical rainforests) [35]. The Amazon basin contains the largest rainforest on Earth, almost 5.8 million Km^2^, and occupies about 40% of South America; more than 60% of the basin is located in Brazil and the rest in Bolivia, Colombia, Ecuador, French Guiana, Guyana, Peru, Suriname and Venezuela.

Over the past decade the positive role of tropical rainforests in capturing large amounts of atmospheric CO_2_ has been demonstrated [36–39]. In response to the increase in atmospheric CO_2_ concentration, tropical forests act as a global carbon sink. Accumulation of carbon in the tropical terrestrial biosphere strongly contributes to slowing the rate of increase of CO_2_ into the atmosphere, thus resulting in the reduction of greenhouse gas effect [5]. Tropical rainforests have been estimated to account for 32–36% of terrestrial Net Primary Productivity (NPP) that is the difference between total forest photosynthesis and plant respiration [40, 41]. In this way tropical rainforests have been acting as a strong carbon sink for decades.

However, over the past years, increased concentrations of greenhouse gases, and especially CO_2_, in the atmosphere have significantly affected the net carbon balance of tropical rainforests, and have warmed the planet substantially driving climate changes through more severe and prolonged heat waves, variability in temperature, increased air pollution, forest fires, droughts, and floods [2, 42]. The role of tropical forests in mitigating climate change is therefore critical. Over the past 30 years almost 600,000 Km2 have been deforested in Brazil alone due to the rapid development of Amazonia, this is the reason why currently the region is one of the ‘hotspots’ of global environmental change on the planet [34]. Deforestation represents the second largest anthropogenic source of CO_2_ to the atmosphere, after fossil fuel combustion [7]. There are many causes of deforestation, including socioeconomic and natural factors, such as clear-cutting for agriculture, ranching and development, unsustainable logging for timber, as well as droughts, fires and degradation due to climate change. About natural causes of forest degradation, in the context of the Amazon, the major agent of change in the forest ecosystem would most likely be decreased dry-season precipitation [43]. Of the 23 global climate models employed by the Intergovernmental Panel on Climate Change (IPCC) in their 2007 report, 50–70% predict a substantial (above 20%) reduction of dry-season rainfall in eastern amazonia under mid-range greenhouse gas emissions scenarios, 40% in central Amazonia and 20% in the west [44]. While annual carbon emissions from fossil-fuel combustion have been continually increasing since 1960s, historical trends of deforestation and associated carbon emissions have remained poorly understood [7, 45, 46]. Recently Song et al., using satellite-data of deforestation rates, derived from changes in tree cover density in the humid tropics, have estimated that between 2000 and 2010, a total of 15.9 ± 2.5 Mha (million ha) forests were lost, which represented 2.6% of the total basin area, or 2.9% of forests in year 2000 [47]. The Brazilian Amazon and the non-Brazilian Amazon lost a total of 12.5 ± 2.0 Mha and 3.4 ± 0.5 Mha forests respectively and over that decade. Brazil was the dominant country in terms of deforested area, which accounted for 79% of the total lost forests [47].

Recently reports by the Brazilian government, the FAO and other previous studies showed a declining trend in the Brazilian Amazon and the entire Amazon basin after 2005 [48–51]. The annual relative share of Brazil’s deforestation changed dramatically over the study period − from the highest of 87% in the year 2004 to the lowest of 54% by the year 2010 [48–51].

Largely driven by Brazil’s efforts to halt deforestation in recent years deforestation rates over the Brazilian Amazon and the entire basin declined significantly in the second half of the last decade, which resulted in greatly reduced carbon emissions [52].

Curbing deforestation in the Brazilian Amazon decreased the Brazilian Amazon’s deforestation contribution to global land use carbon emissions from 17% in the 1990s and early 2000s to 9% by 2010 [53]. An opposite emission trend was observed in the non-Brazilian Amazon; this consisted of various inter-annual variabilities in the Bolivian Amazon, the Colombian Amazon and the Peruvian Amazon. Furthermore, forests of higher-biomass accounted for an increasing portion of the cleared area. According to the Intergovernmental Panel on Climate Change (IPCC), it is necessary to reduce the anthropogenic emissions of CO_2_ to the atmosphere to avoid global warming beyond two degrees [54]. Although tropical deforestation was excluded from the Kyoto Protocol (KP), since 2005 there has been a common effort within the United Nations Framework Convention on Climate Change (UNFCCC) to develop a climate policy approach to deforestation that would compensate tropical nations which reduce carbon emissions from tropical deforestation and forest degradation [55, 56]. The result was a program, known as REDD (“Reducing Emissions from Deforestation and Degradation”) that represents one of the most advanced components of the current round of climate treaty negotiations within the UNFCCC [57, 58].

Reducing fossil fuel emissions remains the key element for stabilizing atmospheric CO_2_; however limiting the emissions from deforestation and degradation of forest represents one of the most cost-effective strategies that can help to stabilize atmospheric CO_2_ levels [59, 60].

## Conclusions

Climate changes affect many physical and biological systems including the immunologic and respiratory systems that are critical to human health, and it is foreseeable that environmental risk factors will have a stronger effect in the coming decades [59–62]. Climate changes interact with and affect air pollution and pollinosis, which in turn increases the frequency and severity of asthma, and affects the clinical expression of allergic disease [1–4]. Climate change affects the timing, dispersion, quantity, and quality of aeroallergens and the distribution and severity of allergic disease. Climate change alters local weather patterns including minimum and maximum temperature, rain precipitation, and storms, all of which affect the burden of allergic disease. A combined approach comprises primary prevention by greenhouse gas mitigation to stabilize the climate, and secondary prevention by clinical intervention to minimize climate change–related increases in asthma and allergic disease [61]. In the future climate changes may depend on how rapidly and successfully global mitigation and adaptation strategies are deployed. The effect of human intervention and efforts to minimize changes in vegetation and aeroallergen exposure remains to be seen.

Reducing air pollution might contribute to lessening the impact of climate change on pollen and thus directly on patients, while recognizing that ozone, the key pollutant associated with climate change, may be the major driver of pollutant/pollen interactions.

What can we do to decrease the effects of environmental factors affecting respiratory allergic diseases? Suggested measures are as follows: encouraging policies to promote access to non-polluting sources of energy; reducing the private traffic in towns and improving public transport; decreasing the use of fossil fuels and controlling vehicle emissions; planting non-allergenic trees in cities, and in this context the proposed implantation of new trees should be evaluated by allergy specialists in order to avoid high allergenic species. Although in this paper the direct impact of the increase in plant growth on allergy has not been dealt with, more studies are needed to assess its contribution to the increase in allergy prevalence.

Many measures to reduce greenhouse gas emissions may have positive benefits for health. These co-benefits will offset at least some of the costs of climate change mitigation and should be taken into account in international negotiations (Table 2). Strategies to reduce climate changes and air pollution are political in nature, but citizen and in particular health professionals and societies must raise their voices in the decision process to give strong support for clean policies on both national and international levels.

**Table 2:** What can we do to reduce the global warming?

• Decreasing use of fossil fuels and controlling vehicle emissions.	
• Reducing the private traffic in towns.	
• Increased use of public transport, cycling and walking.	
• Planting in cities non-allergenic trees.	
• Minimize outdoor activity on days with high pollution.	
• Suggest patients live in remote areas from heavy traffic.	
• Reduction in meat consumption.	
• Two for the price of one: climate change mitigation measures also reduce air pollution.	

## Abbreviations

COPD: Chronic obstructive pulmonary Disease
DEP: Diesel exhaust particulate
EPA: Environmental protection Agency
FAO: Food and agriculture Organization
IPCC: Intergovernmental panel on climate change
NPP: Net primary Productivity
PM: particulate matter
REDD: Reducing emissions from deforestation and degradation
UNFCCC: United nations framework convention on climate change
